# Peptide Tyrosine-Tyrosine Triggers GLP-2-Mediated Intestinal Hypertrophy After Roux-en-Y Gastric Bypass

**DOI:** 10.1007/s11695-022-06328-x

**Published:** 2022-10-27

**Authors:** Gonzalo-Martín Pérez-Arana, Alfredo Díaz-Gómez, Alonso Camacho-Ramírez, Antonio Ribelles-García, David Almorza-Gomar, Manuel Gracia-Romero, José-Arturo Prada-Oliveira

**Affiliations:** 1 Department of Human Anatomy and Embryology, Faculty of Medicine, University of Cadiz, 11003 Plaza Fragela s/n., Cadiz, Spain; 2 Institute for Biomedical Science Research and Innovation (INIBICA), University of Cadiz, 11009 Cadiz, Spain; 3 San Carlos Hospital, Andalusian Health System, 11100 Andalusia, Spain; 4 Surgery Unit, Puerta del Mar University Hospital, University of Cadiz, 11009 Cadiz, Spain; 5 Operative Statistic and Research Department, University of Cádiz, 11003 Cadiz, Spain; 6 Intensive Care Unit, Jerez University Hospital, Andalusian Health System, 11407 Andalusia, Spain

**Keywords:** Type 2 diabetes mellitus, Roux-en-Y gastric bypass, Enterohormones, Peptide tyrosine-tyrosine, Glucose transporter, Pancreas, Intestinal tube, RYGB, Incretines, PYY

## Abstract

**Purpose:**

Intestinal remodeling and adaptation of the alimentary limb after Roux-en-Y gastric bypass (RYGB) play an important role in the pathophysiological events that lead to type 2 diabetes mellitus (T2DM) improvement. Intestinal absorptive loop hypertrophy and growth following surgery have been related to GLP-2 secretion by ileal L-cells. The secretion of peptide tyrosine-tyrosine (PYY) enterohormone after a meal has been proposed as a trigger for ileal secretion of GLP-1. Our aim is to determine the role of PYY as a GLP-2 secretion modulator as an adaptation result in the alimentary limb after RYGB.

**Method:**

We used a non-obese euglycemic rodent model. Circulating glucose, insulin, PYY, and GLP-2 were measured in the experimental and control groups. We used four groups: fasting control, Sham-operated, RYGB-operated (RYGB), and RYGB-operated and treated with BIIE0246 (RYGB + BII). BIIE0246 is a NPY2 receptor antagonist in L-cells. Intestinal glucose transporters and GLP-1 and PYY gut expression and hypertrophy were analyzed after 12 weeks of surgery.

**Results:**

RYGB increased PYY3-36 plasma levels in rats with or without BII treatment. A high-insulin response was observed in the RYGB group but not in the control or RYGB + BII groups. BIIE0246 treatment limited plasma GLP-2 levels. In the alimentary intestinal limb, hypertrophy and SGLT1 and GLUT1 expression appeared to be reduced after RYGB compared to controls.

**Conclusion:**

The postprandial ileal PYY secretion is enhanced after RYGB. This increase mediates GLP-2 release through its binding to the Y2 receptor on L-cells. This mechanism plays a role in alimentary limb hypertrophy after surgery.

**Graphical abstract:**

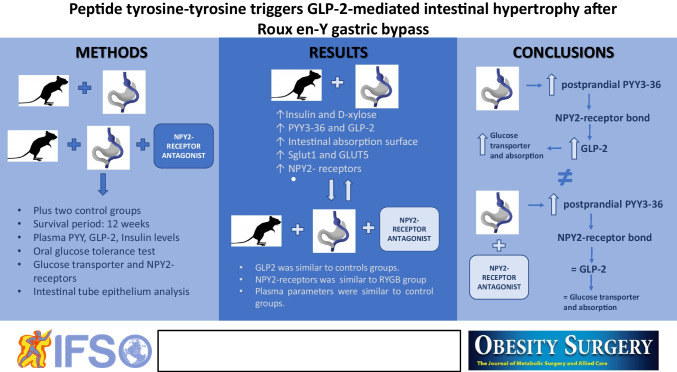

## Background 

Currently, Roux-en-Y gastric bypass (RYGB) is one of the most commonly performed bariatric surgeries [1 ], and it improves sustained glycemic parameters in type 2 diabetes mellitus (T2DM) patients [2 ]. Although many efforts have been made, a large part of the underlying pathophysiological mechanisms remains unknown. However, there is a clear point that has achieved the consensus of authors, which is the key role of the small intestine [3 ]. It has been established that anatomical rearrangement after RYGB induces intestinal remodeling and adaptation of the alimentary limb. These changes produce hyperplasia that increases the glucose and carbohydrate transport capacity of the intestine. The pathway occurs through SGLT1, GLUT1, and GLUT5 intestinal transporter overexpression in animal models and patients [4 ]. This situation is very interesting if we consider the long-term impact of the intestinal glucose flux toward general blood circulation on pancreatic beta cell stimulation and insulin release. This continuing impact on glycemia has been demonstrated as a cause of beta cell population depletion [5 ].

Conversely, hyperplasia of the alimentary limb also implies changes in enteroendocrine cell populations, among which L-cells or K cells secrete GLP-1 and GIP [6 ]. Thus, dumping syndrome or severe postprandial hypoglycemia are some of the most important and frequent complications after RYGB [7 ]. The combination of early elevated plasma GIP and GLP-1 levels after meals has been proposed for an insulinotropic effect that could trigger postprandial hypoglycemia [8 , 9 ]. Therefore, the adaptation and hyperplasia of the small intestine seem to play a basal role in the metabolic changes that surround RYGB surgery.

GLP-2 is also an incretin secreted mainly by ileal L-cells, such as GLP-1 or PYY [10 ]. Early stimulation of L-cells by nutrients has been proposed as an explanation for the resolution of T2DM after RYGB [11 ]. In addition, high GLP-2 plasma levels have been observed after RYGB [12 ], and intestinal absorptive loop hypertrophy and growth following surgery have been related to GLP-2 [13 ]. In this sense, the presence of the Y2 receptor, a member of the NPY receptor family, with high affinity for PYY has been observed in the ileal mucosa nervous efferences [14 ]. Recently, we described the early secretion of PYY after a meal as a trigger for ileal secretion of GLP-1 in diabetic GK rats after RYGB [15 ].

Therefore, we consider it very interesting to study the role of PYY in GLP-2 secretion and gut adaptation after RYGB. For this purpose, we performed RYGB in a rodent model based on healthy Wistar rats. We analyzed the functional and histological parameters related to alimentary limb hypertrophy after surgery.

## Research Design and Methods

### Animals

The animal procedures were approved by the Committee for Ethical Use and Care of Experimental Animals at Cádiz University in accordance with international relevant guidelines and regulations for animal welfare. Twenty-four male Wistar rats weighing 200–220 g, at the age of 10–11 weeks old, were provided and kept at the Experimentation and Animal Production Service of the University of Cádiz (SEPA).

### Experimental Protocol

For hormonal and histological studies, twenty-four Wistar rats were randomly divided into four groups: *n* = 6 fasting control (FC); *n* = 6 Sham-operated rats (Sham); *n* = 6 Roux-en-Y gastric bypass-operated rats (RYGB); and *n* = 6 RYGB-operated rats treated with BIIE0246, a Y2 receptor antagonist (RYGB + BII). The animals were kept for twelve weeks from surgery until sacrifice.

### Surgical Interventions

All groups of rats were subjected to pre- and postsurgical 12-h fasting periods. RYGB-operated animals were anesthetized with continuous infusion of isoflurane (Isoflo, Abbott, USA). A laparotomy of 30 mm in the midline of the abdomen was made. A gastric pouch with a volume of 20% of the normal gastric volume was created. The remnant gastric fundus was anastomosed to the jejunum via end-to-end anastomosis. To this, the jejunum was transected at 14 cm distal from the ligament of Treitz. The biliopancreatic loop was continued with the jejunum at 10 cm of the fundus-jejunum anastomosis; this anastomosis resulted in an end-to-wall anastomosis. The abdominal muscular and skin layers were closed together in one layer using a continuous suturing technique.

Sham-operated animals were anesthetized as above, and a 30-mm incision in the midline of the abdomen was made. The jejunum was transected 400 mm distal to the ligament of Treitz, and a re-anastomosis was performed. The abdominal layers were also closed in one layer using continuous suturing.

### Y2-Receptor Antagonist Treatment

The animals in the RYGB + BMII group were intraperitoneally injected with a solution of BIIE0246 (Techne Corporation, R&D System, Minneapolis, MN, USA) at 10 mg/kg every 48 h from the fifth day after the intervention to the end of the experiment. The other animals in the experiment were injected with saline at the same time.

### Weight Measurement

The weight gain of every animal in the four groups studied was measured every 48 h for 40 days after surgery. Weight gain was expressed as the medium daily difference in grams.

### Oral Glucose Tolerance Test (OGTT) and Insulin Measurement

Ten weeks after surgery, an oral glucose tolerance test (OGTT) was performed in the four experimental groups. A 2 g/kg (20% w/v) d-glucose solution was administered by gavage, and glycemia was measured using a glucometer (Glucocard G-Meter 1810, Menarini Diagnostics, Italy) in blood samples obtained from the tail veins at 0, 30, 60, 90, and 120 min after glucose solution administration.

Ten weeks after surgery, in 12-h-fasted rats from all groups, insulin measurement was performed in blood samples from the tail veins every 10 min for 60 min after glucose solution administration using an ELISA kit (ALPCO Diagnostics, Salem, NH, USA).

### Hormonal Study

Twelve weeks after surgery, a 4 ml/kg, 13.9 kJ/ml mixed meal was administered to 12-h-fasted rats by oral gavage in each group. Subsequently, blood samples were obtained from the rat tails every 15 min for 120 min, added to EDTA tubes containing dipeptidyl peptidase 4 (DPP-4) inhibitor (10 μl/ml blood; Millipore), and centrifuged at 4000 × *g* for 15 min at 4 °C. The plasma was removed, frozen, and stored at − 80 °C. PYY3-36 was assessed by sandwich ELISA kits (Cloud-Clone Corp, USA). GLP-2 was measured using an ELISA kit (Abcam, Cambridge CB4 OFL, UK) according to the manufacturer’s instructions. Finally, the area under the curve (AUC) was calculated by the trapezoidal rule for every parameter to study.

### d-Xylose Absorption Assay

d-Xylose absorption assay was performed in all groups. A d-xylose solution (0.8 g/kg body weight) was administered by oral gavage, and plasma d-xylose levels were measured at 0, 60, 120, and 180 min after loading using a spectrophotometric method, as described by Roe and Rice. The values obtained were expressed as d-xylose mmol/ml of plasma. Areas under the curve (AUCs) were calculated by the trapezoidal rule for each parameter in the study and expressed as plasma d-xylose mmol/ml min^−1^.

### Sacrifice and Tissue Preparation

Animals were sacrificed 12 weeks after surgery according to the Committee for Ethical Use and Care of Experimental Animals at Cádiz University’s instructions by isoflurane overdose. Gut samples were immediately removed, and 1-cm full-thickness segments of duodenum, jejunum, and ileum were harvested and fixed in Bouin’s solution for twelve hours at 4 °C. Later, the tissue samples were dehydrated, embedded in paraffin and cut into serial 10-μm microtome sections.

### Tissue Analysis

For histological analysis, samples of the alimentary limb (gastro-jejunum loop) from the RYGB + BII and RYGB groups were taken. From the Sham and FC rats, the jejunum sample were taken at 20 cm of the Treitz angle. We measured villus height, crypt depth and absorbent mucosae surface in 10 fields of eosin-hematoxylin-stained slices. Values are expressed as length in μm.

In rehydrated sections of intestine, PYY and GLP-2 and NPY2R expression was analyzed by immunostaining using rabbit anti-PYY (Abcam, Cambridge CB4 OFL, UK), rabbit anti-GLP-2 (Abcam, Cambridge CB4 OFL, UK), and mouse anti NPY2R (Antibodies Inc., Davis, CA, 95,617–1560, USA) primary antibodies. To assess the samples, the secondary antibodies used were Alexa 488 anti-rabbit IgG and Alexa 546 anti-mouse IgG (Molecular Probes Inc.). DAPI was used to counterstain nuclei. To determine the positive cell fraction, the number of PYY- or GLP-2-positive cells and intestinal total areas were quantified in 10 fields per condition. The results were noted under randomized conditions and expressed as the numbers of PYY-, GLP-2-, or NPY2R-positive cells/mm [2] of the intestine.

Additionally, in serial rehydrated sections from gut samples of the FC, sham, RYGB, and RYGB + BII rats corresponding to the alimentary limb or the equivalent part of the intestine, glucose transporter expression was analyzed. We used rabbit anti-SGLT1 IgG (Abcam, Cambridge CB4 OFL, UK), rabbit anti-GLUT 1 IgG (Bioss Inc., Woburn, MA, USA) and rabbit anti-GLUT5 IgG (Thermo-Fisher Inc., Burnaby, BC V5C 6S5, Canada). The fluorescent secondary antibody was Alexa 488 anti-rabbit (Molecular Probes Inc.). DAPI was used to counterstain nuclei. Values are expressed as SGLT-1-, GLUT1- or GLUT5-positive area/total intestinal area.

Each histological parameter was measured and noted by a single investigator using a fluorescence microscope with a digital camera and Cell-D image analysis software (Olympus, GmbH. Hamburg, Germany).

### Statistical Analysis

Data are presented as the means ± SEMs. For the AUC, histological and weight gain data analysis, one-way ANOVA followed by Tukey’s/Bonferroni’s post hoc test was conducted using SPSS software, version 21.0. Statistical significance was accepted at *P* < 0.05.

## Results

### Functional Parameters: Weight Gain, OGTT, Insulin Measurement, and d-Xylose Absorption Assay

**Fig. 1:**
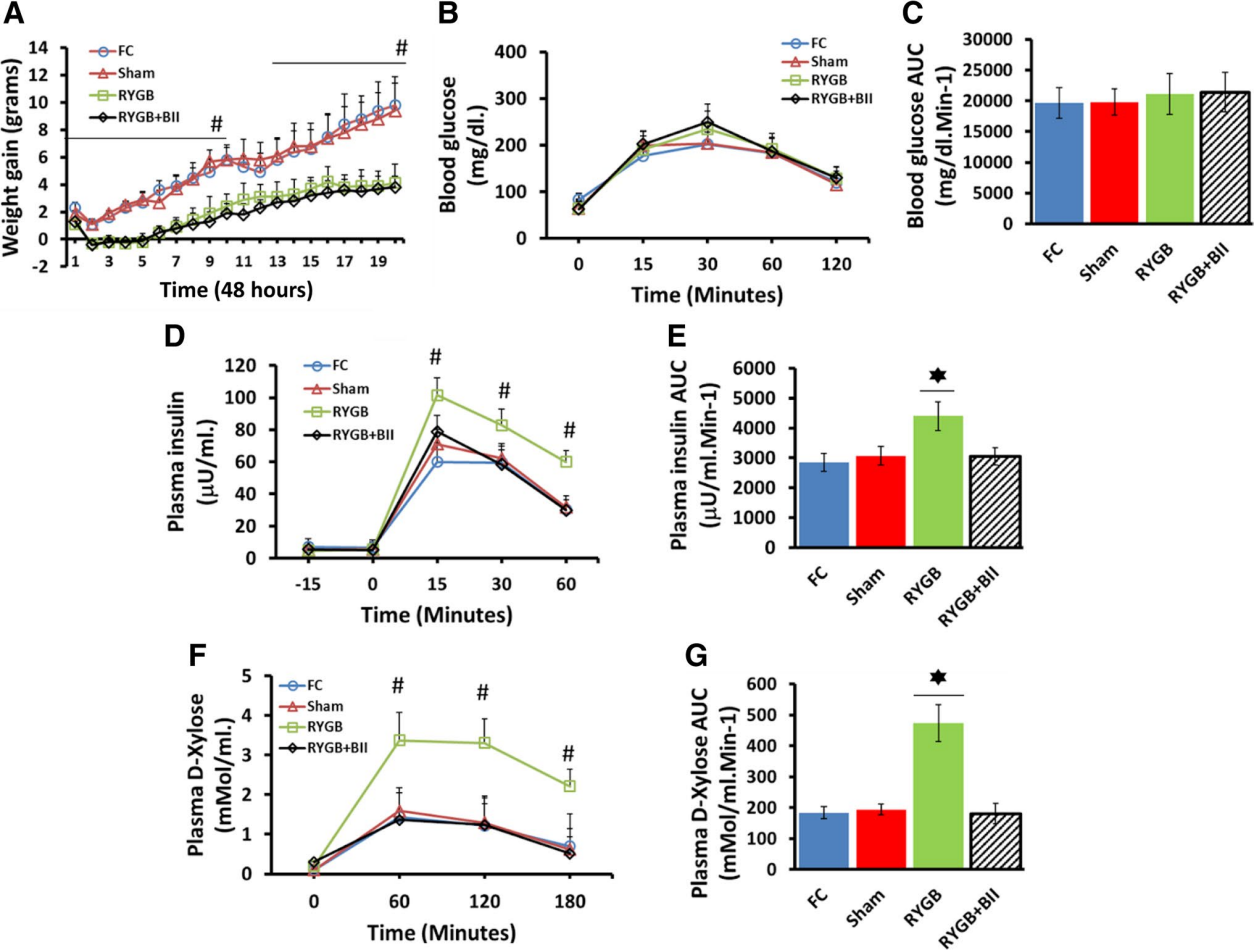
Functional assays. **A** Weight gaining in fasting control (FC, blue line with circles); Sham-operated (Sham, red line with triangles); RYGB-operated (RYGB, green line with squares); RYGB-operated treated with BMS193885 (RYGB + BII, black line with diamonds). Weight is represented as grams on the Y axis over forty days following surgery represented on the X axis. Values are expressed as the mean ± SEM (#*P* < 0.05). **B** Oral glucose tolerance test (OGTT) in *n* = 6 fasting control rats; FC (blue line with circles), *n* = 6 Sham-operated rats; Sham (red line with triangles), *n* = 6 RYGB-operated rats; RYGB (green line with squares), and *n* = 6 RYGB-operated rats treated with BIIE0246; RYGB + BII (black line with diamonds). Glycemia were represented as mg/dl in the Y axis and time after glucose load in the X axis. Values were expressed as mean ± SEM (#*P* < 0.05). **C** OGTT area under curve (AUC) values were presented as mg/dl min^−1^ in the Y axis and expressed as mean ± SEM (*P < 0.05) for each group presented in the X axis. FC (Blue bar), Sham (Red Bar), RYGB (green bar), and RYGB + BII (striped black bar). **D** Plasma insulin in fasting control (FC, blue line with circles); Sham-operated (Sham, red line with triangles); RYGB-operated (RYGB, green line with squares); RYGB-operated treated with BMS193885 (RYGB + BII, black line with diamonds). Plasma insulin levels were represented as μU/ml in the Y axis and minutes after glucose load in minutes in the X axis. Values were expressed as mean ± SEM (#*P* < 0.05). **E** Insulin release area under curve (AUC) values were presented as μU/ml min^−1^ in the Y axis and expressed as mean ± SEM (*P < 0.05) for each group presented in the X axis. FC (Blue bar), Sham (Red Bar), RYGB (green bar), and RYGB + BII (striped black bar). **F** Plasma d-xylose absorption assay insulin, 11 weeks after surgeries, in fasting control (FC, blue line with circles); Sham-operated (Sham, red line with triangles); RYGB-operated (RYGB, green line with squares); RYGB-operated treated with BMS193885 (RYGB + BII, black line with diamonds). Plasma d-xylose levels are represented as mmol/ml in the Y axis versus time after ingestion in minutes in the X axis. Values were expressed as the mean ± SEM (#*P* < 0.05). **G** Plasma d-xylose area under curve (AUC) values were presented as mmol/ml min^−1^ in the Y axis and expressed as the mean ± SEM (*P < 0.05) for each group presented in the X axis

We measured weight gain in the FC, sham, RYGB, and RYGB + BII groups 40 days after surgery. There were no differences between the control groups from the first day to thirty–fourth day. However, the RYGB and RYGB + BII animals showed a low weight gain with respect to the FC and Sham animals at all times (*P* < 0.05#) (Fig. 1A).

Ten weeks after surgery, OGTT was performed on the FC, sham, RYGB, and RYGB + BII groups. Similar curves were described by the four groups. There were no significant differences between glucose tolerance patterns (Fig. 1B). The area under the curve (AUC) was calculated for the four groups. Additionally, no significant differences between any groups were found (Fig. 1C).

Additionally, at the tenth week, insulin secretion was analyzed after SOG. A high plasma insulin secretion pattern appeared in the RYGB animals with respect to the FC, Sham, and RYGB + BII animals (*P* < 0.05#) (Fig. 1D). Insulin secretion AUCs after SOG were also tested, and significantly increased AUC values were found for the RYGB group compared with the control and RYGB + BII groups (*P* < 0.05*) (Fig. 1E).

Finally, a d-xylose absorption assay was performed on the FC, sham, RYGB, and RYGB + BII groups. An increased absorption pattern appeared on the RYGB curve with respect to the FC, Sham, and RYGB + BII animals (*P* < 0.05#) (Fig. 1F). Additionally, a significant increase was observed between the plasma d-xylose AUC from the RYGB rats and controls or the RYGB + BII rats (*P* < 0.05 *) (Fig. 1G).

### Hormonal Study

**Fig. 2:**
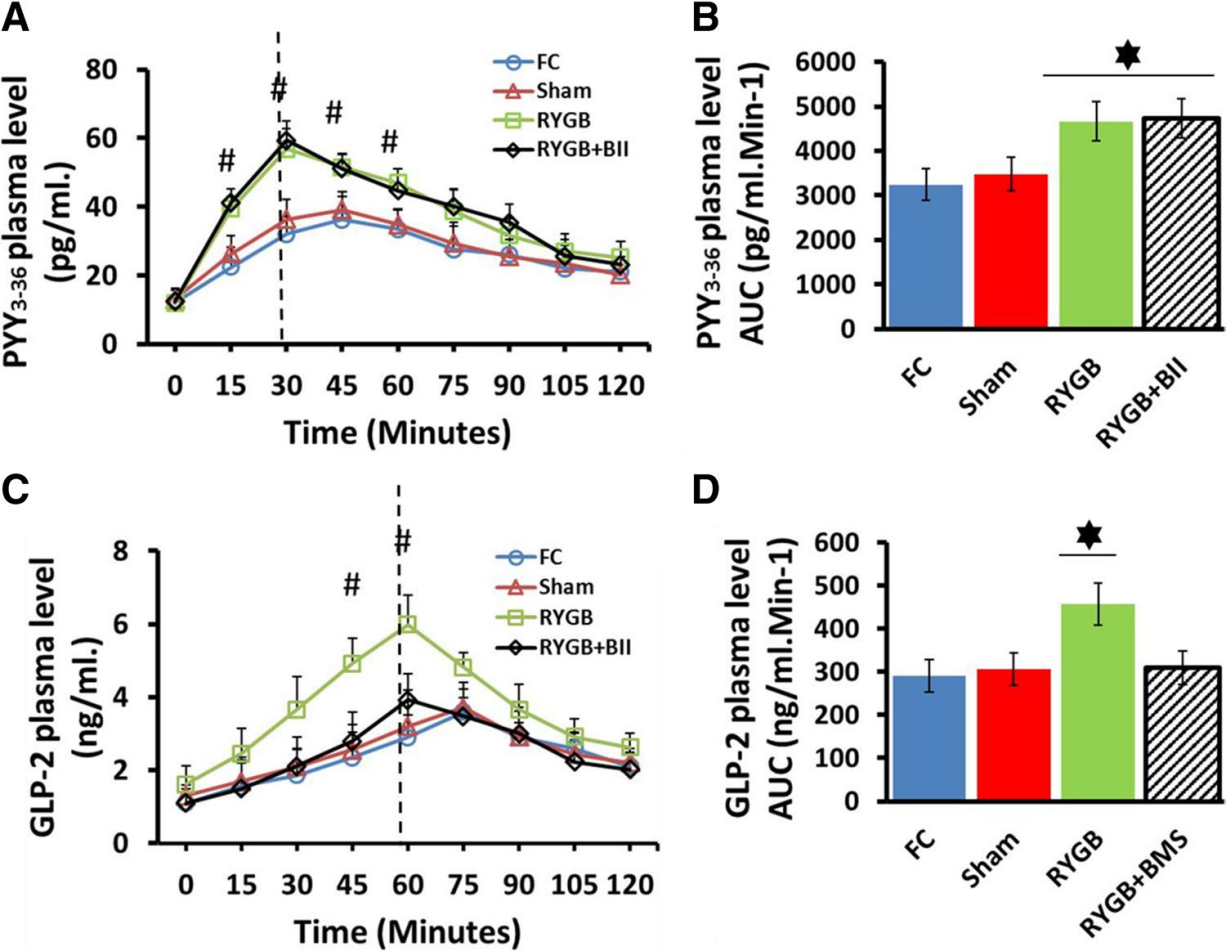
Hormonal study. **A** Plasma PYY3-36 secretion pattern after mixed-meal administration in fasting control (FC, blue line with circles); Sham-operated (Sham, red line with triangles); RYGB-operated (RYGB, green line with squares); RYGB-operated treated with BMS193885 (RYGB + BII, black line with diamonds). PYY3-36 plasma levels were represented as pg/ml in the Y axis and time in minutes after mixed-meal load in the X axis. Values were expressed as mean ± SEM (#*P* < 0.05). **B** Plasma PYY3-36 secretion area under curve (AUC) values were presented as pg/ml min^−1^ in the Y axis and expressed as mean ± SEM (*P < 0.05) for each group presented in the X axis FC, (blue bar), Sham (red bar), RYGB (green bar), and RYGB + BII (Striped black bar). **C** Plasma GLP-2 secretion pattern after mixed-meal administration in fasting control (FC, blue line with circles); Sham-operated (Sham, red line with triangles); RYGB-operated (RYGB, green line with squares); RYGB-operated treated with BMS193885 (RYGB + BII, black line with diamonds). GLP-2 plasma levels were represented as ng/ml in the Y axis and time in minutes after mixed-meal load in the X axis. Values were expressed as mean ± SEM (#*P* < 0.05). **D** Plasma GLP-2 secretion area under curve (AUC) values were presented as ng/ml min.^−1^ in the Y axis and expressed as mean ± SEM (*P < 0.05) for each group presented in the X axis. FC, (blue bar), Sham (red bar), RYGB (green bar) and RYGB + BII (Striped black bar)

We measured the circulating plasma fractions of PYY 3–36 and GLP-2 after oral mixed-meal administration in the four study groups 9 weeks after surgery. As Fig. 2A shows, an enhanced PYY 3–36 secretion pattern appeared after mixed-meal administration in the RYGB-operated rats with or without BIIE0246 treatment compared with the control groups (*P* < 0.05 #). In addition, the PYY_3-36_ AUC value (Fig. 2B) was significantly different and increased in both groups with respect to the controls (*P* < 0.05*).

Additionally, we analyzed GLP-2 plasma levels in each group. Significantly increased GLP-2 plasma levels were detected in the RYGB-operated rats (*P* < 0.05*) but not in the RYGB + BII animals, which showed similar values to those observed in the FC or Sham animals (Fig. 2C). Plasma GLP-2 AUC values also showed high secretion of GLP-2 only by the RYGB group (Fig. 2D) (*P* < 0.05*).

### Gut Histological Study

**Fig. 3:**
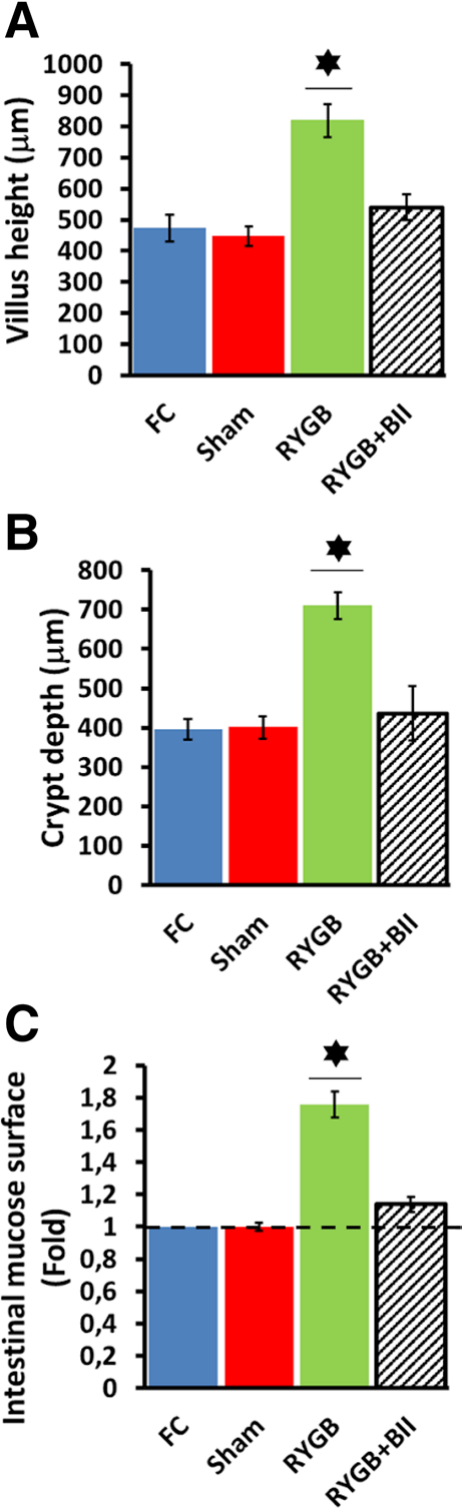
**A** Villus height in gut samples from the alimentary limb or equivalent portion in controls, expressed as μm in the Y axis, as the mean ± SEM (*P < 0.05) for each group to study: FC (blue bar), Sham (red bar), RYGB (green bar), and RYGB + BII (striped black bar). **B** Crypt depth in gut samples from the alimentary limb or equivalent in controls expressed as μm in the Y axis, as the mean ± SEM (*P < 0.05) for each group to study. FC (blue bar), Sham (red bar), RYGB (green bar), and RYGB + BII (striped black bar). **C** Ratio of superficial intestinal mucosa in the Y axis, as average differences since the FC measured value, expressed as the mean ± SEM (*P < 0.05). In the X axis, each group represented FC (blue bar), Sham (red bar), RYGB (green bar), and RYGB + BII (striped black bar)

An increased villus height was observed in alimentary limb samples from the RYGB rats versus all of the other groups 12 weeks after surgery (*P < 0.05), as Fig. 3A shows. Crypt depth was also analyzed, and a strong development of crypts from RYGB alimentary limb samples was observed (*P < 0.05). The FC, Sham, and RYGB + BII groups did not show increased crypt depth development (Fig. 3B). As a result, the absorption surface increased significantly in the RYGB rats with respect to the other groups (Fig. 3C).

### Glucose Transporter, GLP-2, and NPY2R Tissue Expression

**Fig. 4:**
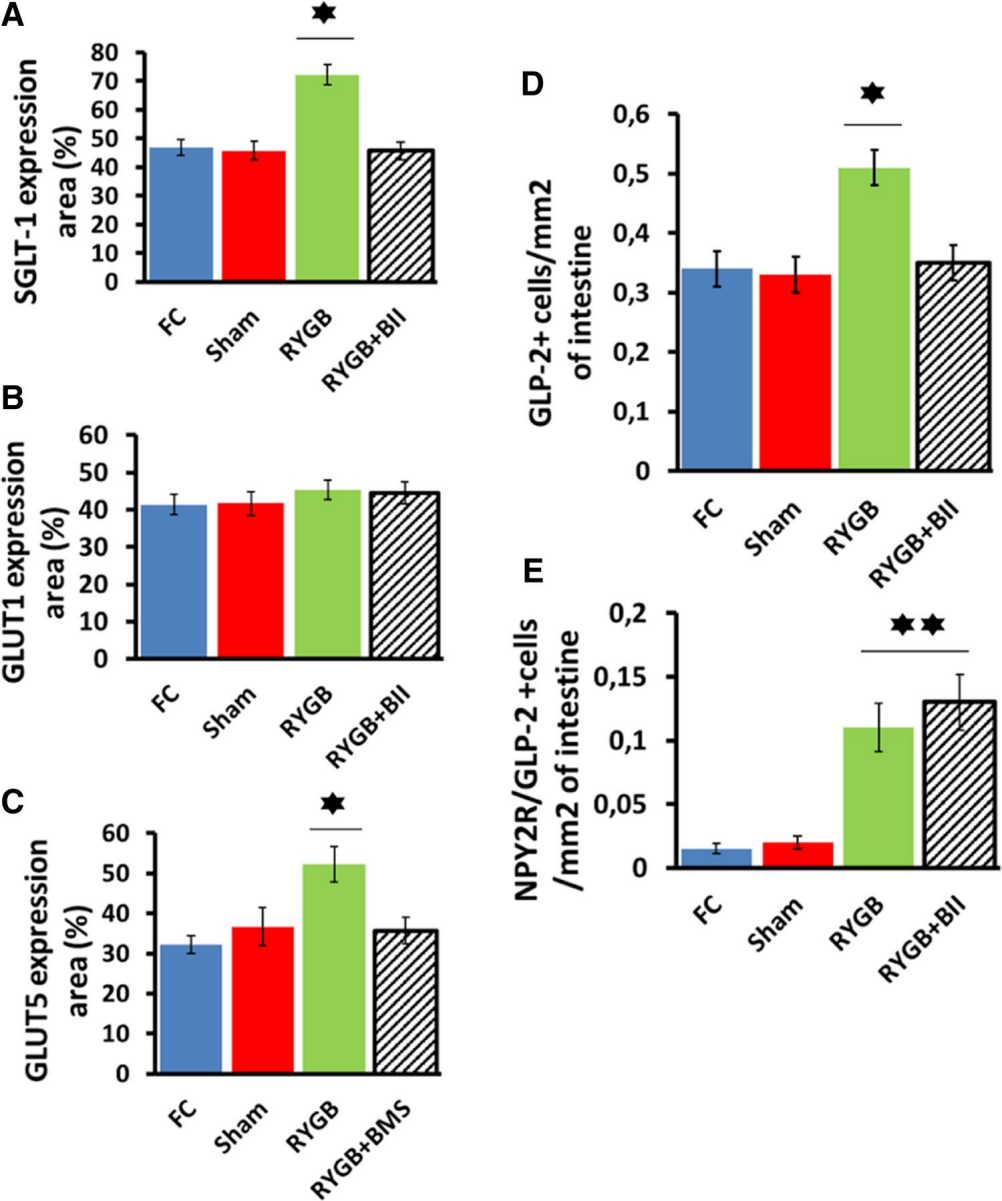
**A** SGLT-1 expression in the alimentary limb or equivalent segment expressed as percentage of SGLT-1 positive area in the Y axis; values were noted as the mean ± SEM (*P < 0.05) for each group to study. FC, (blue bar), Sham (red bar), RYGB (green bar), and RYGB + BII (striped black bar). **B** GLUT1 expression in the alimentary limb or equivalent segment, expressed as percentage of GLUT1-positive area in the Y axis; values were noted as the mean ± SEM (*P < 0.05) for each group to study. FC (blue bar), Sham (red bar), RYGB (green bar), and RYGB + BII (striped black bar). **C** GLUT5 expression in the alimentary limb or equivalent segment expressed as percentage of GLUT5 positive area in the Y axis; values were noted as the mean ± SEM (*P < 0.05) for each group to study. FC (blue bar), Sham (red bar), RYGB (green bar), and RYGB + BII (striped black bar). **D** GLP-2 expression in the alimentary limb or equivalent segment expressed as number of GLP-2-positive cells/mm^2^ of intestine area in the Y axis; values were noted as the mean ± SEM (*P < 0.05) for each group to study. FC (blue bar), Sham (red bar), RYGB (green bar), and RYGB + BII (striped black bar). **E** GLP-2 and NPY2R co-expression in the alimentary limb or equivalent segment expressed as number of doubled GLP-2/NPY2R-positive cells/mm^2^ of intestine area in the Y axis; values were noted as the mean ± SEM (**P < 0.01) for each group to study. FC (blue bar), Sham (red bar), RYGB (green bar), and RYGB + BII (striped black bar)

We measured SGLT-1, GLUT1, and GLUT5 glucose transporter expression in the alimentary limb or the equivalent segments of intestine from each group 12 weeks after interventions. As Fig. 4A and C show, SGLT-1 and GLUT5 tissue expression appeared to be increased in the RYGB animals with respect to the controls (FC and Sham groups) (*P < 0.05). However, the RYGB-operated animals treated with an NPY2R antagonist showed SGLT-1 and GLUT5 expression levels similar to those of the control groups (Fig. 4A to C).

Additionally, GLP-2 and NPY2R expression was measured in the intestine samples of each animal from every group. The data showed a high number of GLP-2-positive cells in the alimentary limb from the RYGB-operated animals without NPY2R antagonist treatment compared to the controls (*P < 0.05). However, there were no differences between the RYGB and RYGB + BII groups, and the GLP-2-positive cell numbers were similar to those of the controls (Fig. 4D).

Finally, NPY2R tissue expression was tested in each group. A large number of doubled GLP-2/NPY2R-positive cells were found in the RYGB-operated animals with or without treatment (**P < 0.01) but not in the control animals (Fig. 4E).

## Discussion

The role of GLP-2 in the growth and adaptation of the alimentary limb following RYGB has long been established [13 ], and our data support it. As Fig. 2C and D show, higher plasma GLP-2 values in the RYGB animals with respect to the controls or the RYGB-operated animals treated with BIIE0246 were found. In addition, we observed a significant development of the intestinal mucosal surface, including greater depth of crypts and greater length of villi in the RYGB group than in the other groups (Fig. 3A–C). This finding agreed with Cavin et al. [4 ] When we paid attention to the hormonal study, we observed an early and higher peak in PYY_3-36_ plasma secretion approximately 30 min after mixed-meal administration. These data were observed not only in the RYGB group but also in the RYGB rats treated with BIIE0246 (Fig. 2A). However, the peak of secretion for the GLP-2 plasma secretion pattern was approximately 60 min. after a mixed meal in the RYGB and RYGB + BII animals (Fig. 2C). In both cases, these peaks were earlier than in the controls. These peaks must be related to anatomical rearrangements after surgery.

These data led us to think about a relationship between PYY_3-36_ and GLP-2 secretion, in which PYY could imply a trigger mechanism for GLP-2 secretion. A similar mechanism was described for PYY and GLP-1 in GK-diabetic rats in previous [15 ]. Considering that NPY2R, the PYY_3-36_ receptor, is expressed in the dorsal vagal complex (DVC) [14 ], we confirmed our hypothesis. First, we used the NPY-2R antagonist BIIE0246 [16 ] to determine the possible dependence of GLP-2 release on PYY_3-36_ secretion. As Fig. 2C and D show, a significant decrease in GLP-2 plasma levels appeared in the RYGB-operated animals treated with the antagonist, even similar to the control groups. This outcome confirmed the relationship between the two events, so the pharma antagonist normalized GLP-2 levels.

We attempted to address the question of whether PYY_3-36_ acts directly on L-cells through an autocrine mechanism or whether it acts through the remaining nervous efferences from the DVC to the ileum. Thus, we determined the expression location of NPY2R in the alimentary limb, or equivalent intestinal segment, using an anti NPY2R antibody. Surprisingly, many cells showed NPY2R and GLP-2 double labeling in contrast to the exclusive presence of NPY2R in enteric neurons proposed thus far [16 ]. This finding suggested that the L-cells themselves were the targets of PYY_3-36_ in RYGB-operated animals because the number of double-labeled cells was practically nonexistent in the FC and Sham animals (Fig. 4E). These data cannot exclude the exclusive activity of PYY_3-36_ on L-cells throughout DVC terminals. The coexistence of both activation mechanisms could be possible.

Attending to the observations in Fig. 4D and E, other explanation can be expressed. The direct autoactivation of L-cells themselves was an amplification mechanism for the PYY_3-36_ effect on GLP-2 secretion in response to the need for fast gut adaptation after surgery. This idea is supported by the finding of NPY2R in the nervous efferences of the gastrointestinal system but not in epithelial or endocrine cells always found in nonoperated animals or patients [17 , 18 ]. Moreover, this double expression of NPY2R and GLP-2 was negligible in the controls, as Fig. 4E shows. An increased number of GLP-2-positive cells appeared only in the RYGB rats without NPY2R antagonist treatment (Fig. 4D).

When we focused on the effect of PYY_3-36_ on alimentary limb mucosal remodeling, we observed a double intestinal mucosa amplification mechanism. We observed a higher villus height and deeper crypts in the RYGB animals (Fig. 3A to C). Additionally, there was a significant increase in the expression of glucose transporters, such as SGLT1 and GLUT5 (Fig. 4A and C). These factors led to intestinal hyperfunctionality, with a high absorption capacity. This outcome was confirmed with the d-xylose assay (Fig. 1F and G) and seemed to be in accordance with previous works [4 , 5 ]. In this way, the high glucose flux from the alimentary limb to the blood circulation could explain the strong insulin response obtained after OGTT in the RYGB animals but not in the other groups (Fig. 1D and E), including the RYGB + BII group, which exhibited limited glucose transporter overexpression.

We think that our findings are relevant if we take account the high percentage of diabetes relapse 5 years after RYGB described in several studies [1 , 19 , 20 ]. The hypertrophy of the alimentary limb and the over expression of many glucose transporter molecules within it has widely reported in animals and patients after RYGB [4 , 21 ]. Also, a previous work has related the increased glucose uptake in the alimentary limb with the long-term beta cell mass depletion and an insulin response failure in rodent models [5 ] to understand the pathophysiological mechanism underlying the gut hypertrophy after RYGB becomes important. The description of the regulatory role of PYY within this mechanism provides us with a therapeutic target for the development of new pharmacological agents, such as inhibitors or selective anti-PYY antibodies. This pharmacological approach could prevent the phenomenon, limiting the excessive glucose flow from the alimentary limb to the bloodstream. This limitation would protect to the long-term beta cell exhaustion and diabetes relapse after surgery.

Summarizing all of these data, the histological and functional modifications after RYGB were abolished by treatment with the BIIE0246 NPY2R antagonist. Currently, many efforts are directed at limiting intestinal glucose transporter activity, especially SGLT-1, in T2DM [22 ]. These efforts included using dual inhibitors for SGLT1/2, such as sotaglifozin or licoglifozin [23 , 24 ]. However, the results have not yet been convincing [25 ]. The use of these inhibitors to prevent the problem of long-term T2DM relapse after RYGB [26 ] is not clear or conclusive.

Moreover, the combination of an initial high glucose excursion due to SGLT-1 overexpression in the intestine and GLP-2 secretion by L-cells seems to be underlying postprandial hypoglycemia syndrome, attending to the glucagonotropic effect of GLP-2 [27 , 28 ]. We propose new knowledge about the mechanism that regulates the adaptation of the intestinal tube to surgery. The importance of these findings lies in their providing a new target to limit the excessive glucose excursion derived from hypertrophy of the alimentary limb. This subsequent glucose transporter overexpression and excessive L-cell activity would constitute the basis of the long-term failure of RYGB. Much work remains to be done on this topics, but it could be an interesting starting point to avoid some of the main problems that appear after RYGB surgery. This work must be related to the use of pharmacological substances to sustain the equilibrium of T2DM improvement homeostasis in the long term.

## Data Availability

All the data supporting the results and critical resources will be available at the institutional repository of the University of Cadiz (http://hdl.handle.net/10498/27288).
